# Key features in telehealth-delivered cardiac rehabilitation required to optimize cardiovascular health in coronary heart disease: a systematic review and realist synthesis

**DOI:** 10.1093/ehjdh/ztad080

**Published:** 2024-01-05

**Authors:** Victor M Gallegos-Rejas, Jonathan C Rawstorn, Robyn Gallagher, Ray Mahoney, Emma E Thomas

**Affiliations:** Centre for Online Health, The University of Queensland, Ground Floor Building 33, Princess Alexandra Hospital, Woolloongabba, QLD 4102, Australia; Centre for Health Services Research, The University of Queensland, Brisbane, Ground Floor Building 33, Princess Alexandra Hospital, Woolloongabba, QLD 4102, Australia; Institute for Physical Activity and Nutrition, School of Exercise and Nutrition Sciences, Deakin University, Burwood, VIC, Australia; Susan Wakil School of Nursing and Midwifery, The University of Sydney, Western Ave, Camperdown, NSW 2050, Australia; CSIRO Health & Biosecurity, Australian e-Health Research Centre, Surgical, Treatment and Rehabilitation Service—STARS Level 7, 296 Herston Rd, Herston 4029, Australia; School of Public Health, The University of Queensland, Brisbane, Australia; Centre for Online Health, The University of Queensland, Ground Floor Building 33, Princess Alexandra Hospital, Woolloongabba, QLD 4102, Australia; Centre for Health Services Research, The University of Queensland, Brisbane, Ground Floor Building 33, Princess Alexandra Hospital, Woolloongabba, QLD 4102, Australia

**Keywords:** Telehealth, Cardiac rehabilitation, Realist review, Secondary prevention, Systematic review

## Abstract

Telehealth-delivered cardiac rehabilitation (CR) programmes can potentially increase participation rates while delivering equivalent outcomes to facility-based programmes. However, key components of these interventions that reduce cardiovascular risk factors are not yet distinguished. This study aims to identify features of telehealth-delivered CR that improve secondary prevention outcomes, exercise capacity, participation, and participant satisfaction and develop recommendations for future telehealth-delivered CR. The protocol for our review was registered with the Prospective Register of Systematic Reviews (#CRD42021236471). We systematically searched four databases (PubMed, Scopus, EMBASE, and Cochrane Database) for randomized controlled trials comparing telehealth-delivered CR programmes to facility-based interventions or usual care. Two independent reviewers screened the abstracts and then full texts. Using a qualitative review methodology (realist synthesis), included articles were evaluated to determine contextual factors and potential mechanisms that impacted cardiovascular risk factors, exercise capacity, participation in the intervention, and increased satisfaction. We included 37 reports describing 26 randomized controlled trials published from 2010 to 2022. Studies were primarily conducted in Europe and Australia/Asia. Identified contextual factors and mechanisms were synthesized into four theories required to enhance participant outcomes and participation. These theories are as follows: (i) early and regular engagement; (ii) personalized interventions and shared goals; (iii) usable, accessible, and supported interventions; and (iv) exercise that is measured and monitored. Providing a personalized approach with frequent opportunities for bi-directional interaction was a critical feature for success across telehealth-delivered CR trials. Real-world effectiveness studies are now needed to complement our findings.

## Graphical Abstract

**Graphical Abstract:**
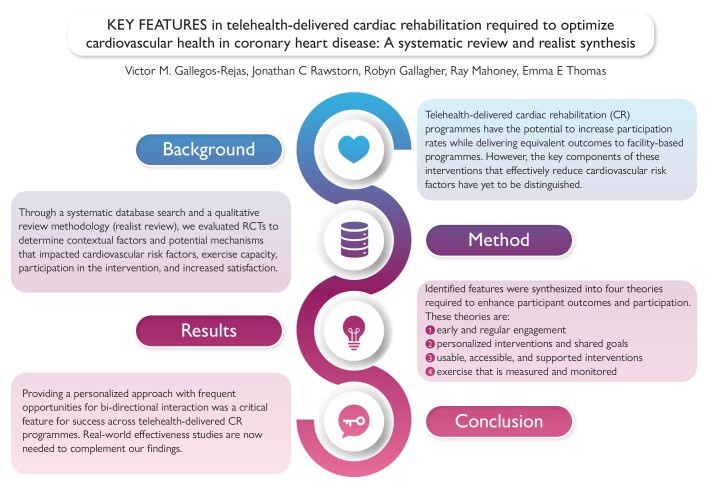

## Introduction

Coronary heart disease (CHD) is a major cause of death and disability globally.^1–3^ Secondary prevention programmes such as cardiac rehabilitation (CR) are a level 1A intervention for CHD management in multiple guidelines internationally.^4,5^ This multicomponent intervention comprises exercise training and lifestyle change education, including physical activity, nutrition, medication compliance, and stress management.^6,7^ Cardiac rehabilitation has been proven to reduce future cardiovascular events and disability,^8–10^ promote healthy behaviours, and enhance health-related quality of life.^6^ Improved clinical outcomes underpin reductions in intervention-related costs to the health system.^2,6,8,11^ Despite these benefits, participation and completion rates remain low; on average, only 30% of eligible participants attend globally.^9,10,12–14^

Barriers, such as transportation issues, lack of facilities nearby, and work and/or carer commitments, have negatively impacted participation in facility-based CR programmes.^15^ Moreover, public health response to the COVID-19 pandemic forced the temporary closure of many facility-based CR programmes, which exacerbated participation barriers with an ultimate reduction in service delivery.^16,17^ Consequently, CR providers have been strongly motivated to test out alternate CR delivery modes to enable participants to continue to access care.^18^

Telehealth-delivered CR is a sound strategy to overcome access barriers and ultimately increase patient participation.^7,16,19^ This intervention uses telecommunication technologies to deliver CR services at home or close-to-home settings.^7,12,16,19,20^ Telehealth-delivered CR uses various technologies, including mobile apps, telephone calls, text messaging, and videoconferencing, to provide rehabilitation care.^16^ Telehealth-delivered CR has also been proven to be safe when based at a patient’s home^21^ and as cost effective as hospital-based programmes.^22^ Multiple meta-analyses of randomized clinical trials have demonstrated that telehealth-delivered CR can result in equal or better cardiovascular outcomes when compared with facility-based programmes.^7,12,20,23,24^ Additionally, providing a telehealth-delivered CR option can also result in higher satisfaction^12^ and increases participation rates.^2,7^

While telehealth-delivered CR can provide equivalent effects on secondary prevention outcomes as compared with facility-based interventions, certain enablers are needed. For designers and implementers of telehealth-delivered CR programmes, it is currently not clear what the key ‘ingredients of success’ are to ensuring optimal outcomes for their telehealth-delivered option. Consequently, further analysis is required to elucidate the key features and mechanisms of successful telehealth-delivered CR programmes. Author E.E.T. had previously conducted a realist review on remote patient monitoring (RPM) interventions,^25^ which included a large heart failure population (15 studies). As such to ensure a novel contribution to the literature, this review excluded heart failure studies and focused on telehealth use within the predominant CR population—CHD.

Therefore, the aim of this study was to use a realist approach^26–28^ to (i) identify features of telehealth-delivered CR that improved secondary prevention outcomes, exercise capacity, participation, and satisfaction in clinical trials within the CHD population and (ii) develop recommendations for future telehealth use within this population.

## Methods

### Protocol and registration

A systematic literature search was conducted in accordance with the Preferred Reporting Items for Systematic Reviews and Meta-Analyses (PRISMA) guidelines.^29^ A protocol was published on the PROSPERO International Prospective Register of Systematic Reviews (#CRD42021236471).

### Realist synthesis approach

We adopted a realist approach,^26^ which uses published evidence reports to answer questions regarding how and why complex health interventions work (or not) in a particular context or setting.^26^ A realist approach^26–28^ provides a better understanding of the relationships between context and outcomes surrounding telehealth-delivered CR implementation in clinical practice,^30^ especially when comparing varied contexts, though the iterative process required can be quite time consuming and subjective. The goal of a realist synthesis is not to aggregate trial measures into a summary figure or a final judgement but, in this case, more about understanding features of CR programmes that lead them to succeed or fail. The meta-analysis by Ramachandran *et al.*^31^ establishes the effectiveness of telehealth-delivered CR; however, further examination was required regarding how and in what contexts it leads to these successful outcomes. We explored the mechanisms by which telehealth-delivered CR impacts secondary prevention outcomes [including body mass index (BMI), lipid profile, smoking cessation, and physical activity], exercise capacity, and participation rates using a context–mechanism–outcome (CMO) configuration. Our findings were reported following the Realist And Meta-narrative Evidence Syntheses: Evolving Standards (RAMESES).^32^

### Search strategy

This systematic review searched for randomized controlled trials (RCTs) to provide a clear understanding of the features impacting secondary prevention outcomes among clinical interventions, which leveraged the level of evidence of this study design methodology. Due to the recent evolution of telehealth technologies, we included studies published within the last decade (January 2010 to September 2022). In February 2021, we initially searched four electronic databases: PubMed (MEDLINE), Embase (OvidSP), Scopus, and CINAHL (EBSCOhost), as well as RCTs published in the ClinicalTrial.gov database. To ensure the currency of information, the search was updated in September 2022. The search strategy combined terms related to telehealth, CR, telerehabilitation, and CHD. The complete PubMed search strategy is available in Supplementary material online, *Table S1*.

### Inclusion criteria

We included peer-reviewed RCTs primarily focused on telehealth-delivered CR delivered to patients with CHD (e.g. post-myocardial infarction, post-angioplasty, and stable angina) and compared this intervention to facility-based CR or usual care. When studies targeted multiple cardiac conditions, the CHD arm was considered for inclusion. Based on the definitions described by Rawstorn *et al.*^24^ and Hwang *et al.*,^33^ studies were included if a telehealth-delivered CR programme included at least 50% patient–provider contact using communication technologies. This information was obtained from the methods section of each selected paper.

We excluded non-randomized trials, studies primarily targeting congenital heart diseases, heart failure, arrhythmia, or valvulopathy as conditions, and study protocols or conference proceedings. Studies that did not provide a telehealth-dominant arm were excluded.

### Study selection

Two reviewers (V.M.G.-R. and E.E.T.) independently screened titles and abstracts using the systematic review management tool Covidence (https://www.covidence.org/) and were blinded to each other’s selections. Disagreements were discussed between these reviewers (V.M.G.-R. and E.E.T.) until a consensus was reached. The full texts of potentially relevant papers were retrieved and assessed for inclusion or exclusion by one reviewer (V.M.G.-R.), with any concerns discussed with the second reviewer (E.E.T.).

### Data extraction

Data extracted from eligible articles included (i) author; (ii) country of origin; (iii) demographic characteristics of participants; (iv) details of the intervention such as exercise prescription (including duration and frequency) and the inclusion of feedback or tailoring to participant needs; (v) clinical outcomes (e.g. exercise tolerance or functional exercise capacity); and (vi) intervention outcomes (participation rates among participants enrolled, acceptability, and intervention uptake), and, based on the realist review methodology,^26^ we collated authors’ interpretation on reasons or mechanisms for the reported conclusions.

### Quality assessment

The quality of the included studies was assessed using version two of the Cochrane risk-of-bias tool (RoB 2).^34^ One researcher (V.M.G.-R.) completed the quality appraisal independently, which included the assessment of sequence generation, allocation concealment, blinding of outcome assessors, blinding of participants, incomplete data, selective outcome reporting, and other sources of bias. None of our articles was excluded based on the quality assessment score.

### Evidence synthesis

Extracted data were recorded using a Microsoft Excel spreadsheet and uploaded to NVivo12® for thematic analysis. Initial coding was performed to identify the authors’ perceptions of factors that influenced the study outcomes. Identified factors were mapped onto four outcomes: (i) secondary prevention outcomes (including BMI, blood pressure, lipid profile, smoking cessation, mood, and physical activity), (ii) exercise capacity, (iii) participation, and (iv) satisfaction. These configurations generated explanations about the contexts (C) and interactions with underlying mechanisms (M) influencing the telehealth-delivered CR outcomes (O).^26^ First, CMO configurations facilitated the development of theories proposing mechanisms about how specific patterns of contexts and outcomes occurred at the intervention level. Then, configurations were refined to explain how mechanisms work with the identified pattern of context and outcomes. Data synthesis was undertaken by one reviewer (V.M.G.-R.), and it was shared and discussed with a second reviewer (E.E.T.) to ensure the appropriateness of the proposed theories. Synthesis of findings was also discussed with all the co-authors to verify the consistency of findings.

## Results

### Study selection

**Figure 1:**
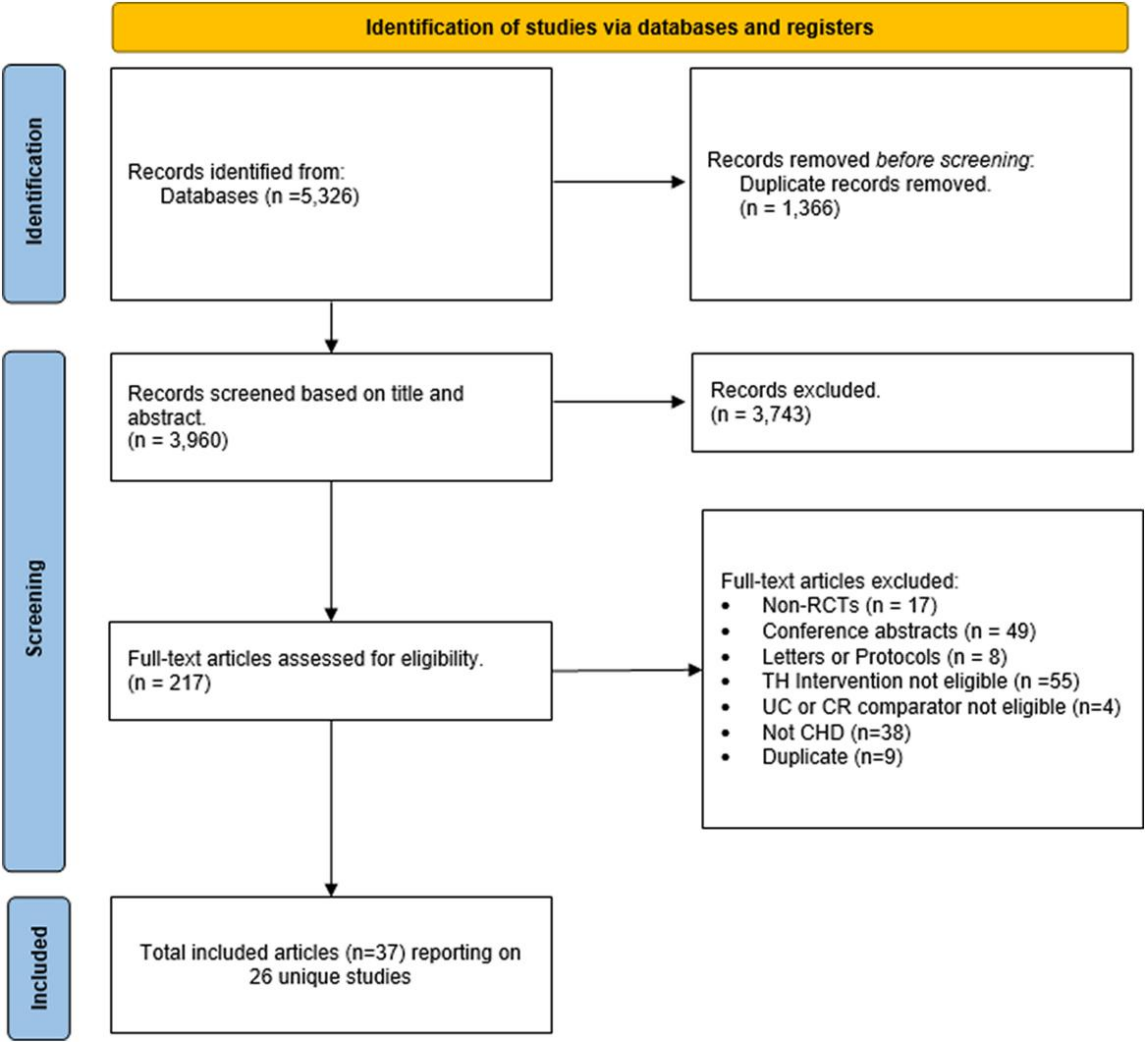
Preferred Reporting Items for Systematic Reviews and Meta-Analyses flow diagram representing the number of search records screened, included, or excluded at each stage of study selection. CHD, coronary heart disease; CR, cardiac rehabilitation; RCT, randomized controlled trial; TH, telehealth; UC, usual care.

We identified 5326 records after the primary search. After duplicate removal, screening occurred on 3960 records. Among 217 full-text articles retrieved and assessed for eligibility, 37 reports describing 26 unique studies were included (*Figure 1*).

### Study characteristics

Included studies were primarily conducted in Europe (*n* = 12, 46%), followed by Australia/Asia (e.g. Australia, China, and Korea; *n* = 11, 42%), and North America (*n* = 3, 12%). Sample sizes ranged from 28^35^ to 710,^36^ with at least 10 people in the intervention arm.^37^ The majority of studies reported >70% male participants (*n* = 16, 62%). The mean age of participants varied from 54^38^ to 72 years old. Seventeen studies (65%) were conducted in metropolitan areas. Telehealth-delivered CR programmes were mainly compared with usual care (i.e. facility-based care and secondary prevention outpatient programme; *n* = 16, 62%), followed by only non-telehealth CR programmes (i.e. regular follow-up medical appointments; *n* = 9, 35%), and compared with usual care combined with non-telehealth CR^39^ (*n* = 1, 3%). Additional characteristics of included studies are presented in Supplementary material online, *Table S2*.

### Study quality

All 26 studies were at risk of bias due to a lack of blinding of participants or treatment delivery personnel. However, blinding participants or personnel was not possible due to the nature of the interventions. On the other hand, more than half (*n* = 17, 65%) reported having included blinding for outcome assessors. In one-fifth of the studies (*n* = 5, 19%), incomplete outcome data were a source of bias, primarily due to attrition bias among study participants. Studies consistently reported secondary prevention outcomes, exercise capacity, participation, and satisfaction rates. Risk of bias assessment is provided in Supplementary material online, *Figure S1*.

### Intervention characteristics

#### Modality of delivery

The most frequently used telehealth technologies were purposively designed applications (either mobile or web apps) that remotely collected and transmitted data from the patient to a health care provider^40^ (*n* = 15, 58%), web-based applications for education support (*n* = 6, 23%), and either text messaging or phone call alone (*n* = 5, 19%).

#### Intervention targets

Half of the included studies (*n* = 13, 50%) only targeted the exercise component of CR. Five studies (19%) targeted exercise and education components, while eight (31%) provided exercise, education, and psychological support.

#### Intervention provider

Seven studies provided nurse-led intervention (*n* = 7, 27%). Three studies (*n* = 3, 12%) described the role of the physiotherapist as the main care provider. One study (*n* = 1, 8%)^41^ reported the exercise physiologist leading the research team and providing care. Multidisciplinary teams, including cardiologists, general practitioners, nurses, physiotherapist, and psychologists, were reported across four studies (*n* = 4, 15%).

#### Intervention dose

Intervention duration varied from 6 weeks (*n* = 3, 12%) up to 24 weeks (*n* = 10, 39%). Thirteen studies (*n* = 13, 50%) provided at least one exercise session per week up to three sessions weekly with at least one counselling or follow-up session per week.

#### Tailoring

Tailored feedback, defined as the information and guidance delivered by clinicians to participants promoting behavioural change and sharing their assessment of patient performance in the CR programme,^4,42^ was provided in more than half the included papers (*n* = 18, 69%). Supplementary material online, *Table S2* provides a summary of intervention characteristics.

#### Modifications

No studies reported on deviations from the intended intervention.

### Study outcome characteristics

#### Secondary prevention outcomes

Most studies reported secondary prevention outcomes that were either non-inferior to the control group or favoured the telehealth group. Seven studies (27%) reported improved secondary prevention outcomes favouring the telehealth group compared with facility-based CR. Twelve studies (46%) reported positive secondary prevention outcomes favouring the telehealth-delivered CR group compared with usual care. Six studies (23%) reported no changes in secondary prevention outcomes, and no studies reported decreased exercise capacity in the telehealth-delivered CR group.

These results included increased physical activity levels (*n* = 7, 27%) or exercise capacity (*n* = 12, 46%) measured by VO_2_peak, 6-min walk distance/test, or metabolic equivalent of tasks (METs). Other secondary prevention outcomes, such as smoking cessation, lipid profile, and BMI, were non-inferior to the intervention group. Of the 19 studies that measured blood pressure, 11 (58%) reported a non-inferior change,^19,35,41,43–50^ six favoured the intervention group (32%),^36,51–55^ and two^56,57^ (11%) favoured the control group. Eight studies measured anxiety and/or depression.^39,43–45,47,48,50,58^ Three studies reported a reduction in anxiety in the telehealth group^45,48,59^; the remaining reported no change.

#### Participation and satisfaction

This study defined participation as the reported proportion of people in a clinical trial setting who attended at least 50% of either the education or exercise sessions while the study was conducted. Ten studies (38%) reported higher participation in the telehealth-delivered CR group than the facility-based or usual care group. Eight studies (*n* = 8, 31%) reported unmodified participation rates in the telehealth-delivered CR group compared with either facility-based interventions or usual care. Similarly, eight studies (*n* = 8, 31%) did not report modification in their participation rates when comparing the telehealth-delivered CR group vs. the control.

Regarding satisfaction rates (defined as the participant’s self-reported feeling of contentment with the process of care and its outcomes), six (*n* = 6, 23%) studies reported higher satisfaction among participants in the telehealth-delivered CR group as compared with facility-based or usual care. In contrast, two studies^56,60^ (*n* = 8%) reported lower satisfaction rates across their participants in the intervention group compared with those undergoing facility-based programmes.

### Realist synthesis

#### Factors that impacted telehealth-delivered cardiac rehabilitation outcomes

**Figure 2:**
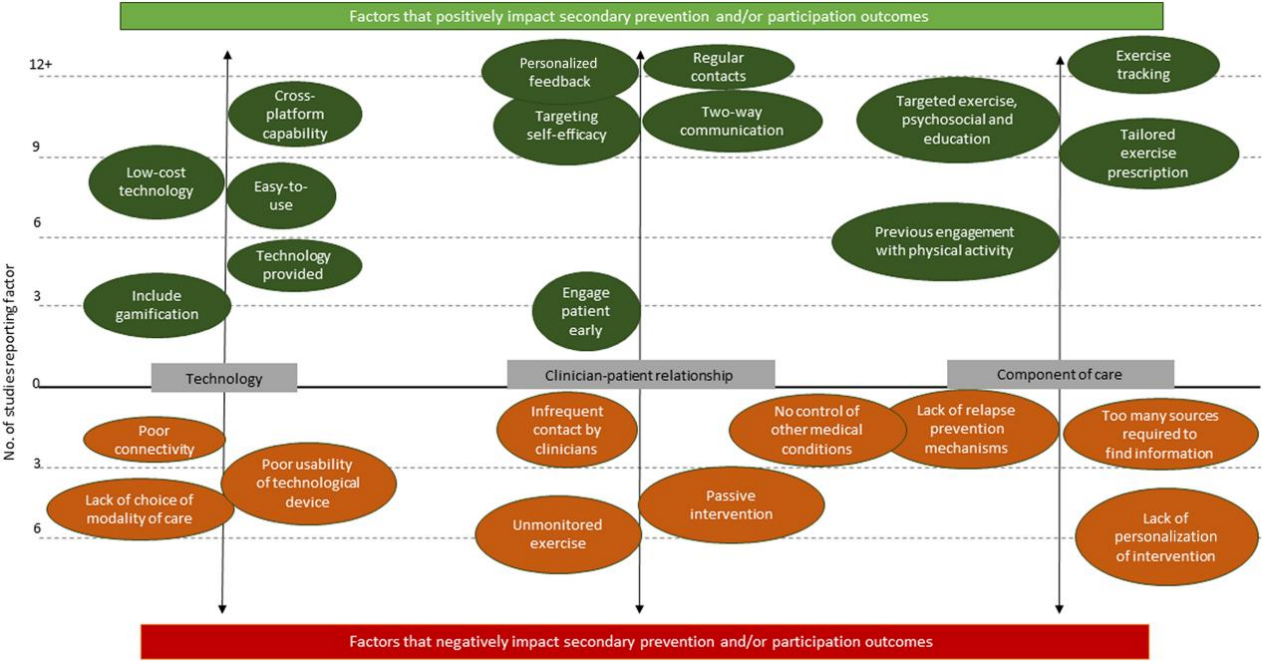
Identified factors that either positively or negatively impacted telehealth-delivered cardiac rehabilitation outcomes.

Our analysis identified 29 factors that either positively (17 factors) or negatively (12 factors) affected telehealth-delivered CR outcomes in clinical trials. Factors were details of how the intervention or programme was implemented (e.g. if smartphones were provided to participants) or characteristics of the programme itself (e.g. if there was weekly contact with participants) that appeared to impact CR outcomes compared with if those factors were not present or were different. Factors were identified and then mapped onto four outcomes: (i) secondary prevention outcomes (including BMI, lipid profile, smoking cessation, and physical activity), (ii) exercise capacity, (iii) participation, and (iv) satisfaction. Due to the large number of factors, they were organized into three groups according to what aspect of the programme they related to, in order to make it easier to plan how they may be applied in the future: (i) technology related, (ii) components of care, and (iii) clinician–participant relationship (see *Figure 2*; Supplementary material online, *Table S3*).

Our analysis identified 12 factors that appear to negatively influence telehealth-delivered CR outcomes, including participation and satisfaction (see Supplementary material online, *Table S4*). Perceived lack of usefulness of the intervention appeared as the leading cause for low participation and satisfaction rates. Interventions lacking bi-directional interaction between participants and clinicians (passive interventions) reported low participation rates since interaction was crucial for building trust and acceptability. Additional factors limiting participation and satisfaction were lack of technological support and poor usability of the technological device.^43,56^

#### Realist theories to improve secondary outcomes, participation, and satisfaction in telehealth-delivered cardiac rehabilitation trials

**Figure 3:**
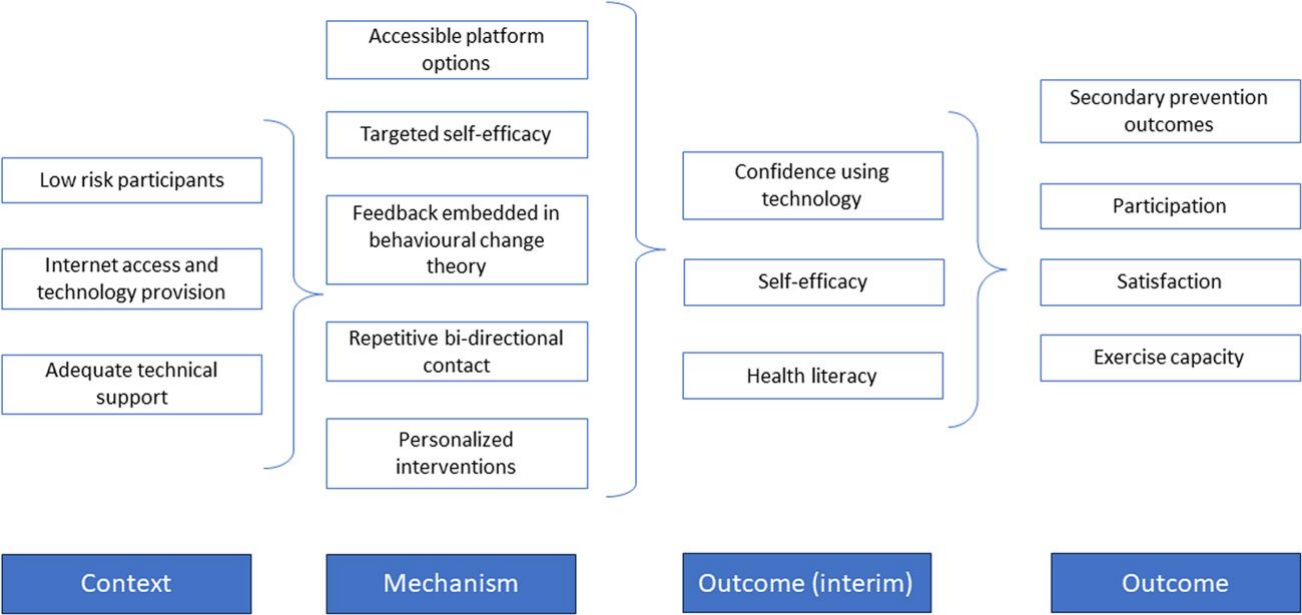
Context–mechanism–outcome theories required to optimize telehealth-delivered cardiac rehabilitation outcomes.

Telehealth-delivered CR outcomes and contextual factors interacted through several underlying mechanisms. To explain these interactions, we developed a CMO configuration (*Figure 3*) described in four theories explaining the mechanisms of action for the success of telehealth-delivered CR interventions: Theory 1—engaging early and regularly; Theory 2—personalizing interventions with an opportunity to develop bi-directional interaction and shared goals; Theory 3—usable and accessible telehealth technologies; and Theory 4—measuring and monitoring exercise.

#### Realist theories to improve secondary outcomes, participation, and satisfaction in telehealth-delivered cardiac rehabilitation trials

##### Theory 1: early and regular engagement

Early engagement is defined as clinician–patient contact during an inpatient stay or within 1 week after hospital discharge to initiate or introduce the option of telehealth-delivered CR.^45^ Participation tended to be higher when participants received early engagement, and it was reported to promote higher participation rates and improved measurements of depression and anxiety among participants in telehealth-delivered CR.^45,51^ Lee *et al.*^45^ reported that providing a personalized CR programme from early stages was safe and effective. This strategy appeared to reduce anxiety and increase exercise capacity, improving health-related quality of life, participation, and satisfaction rates.^45^ Similarly, Yudi *et al.*^51^ highlighted the significant contribution of early engagement to goal achievements in exercise capacity and participation. Their results were influenced by early familiarization with telehealth technologies (e.g. initiating telehealth-delivered CR programme on hospital discharge), which allowed participants to develop confidence in using the intervention.^51^

Telehealth-delivered CR trials providing repetitive contact with participants were more likely to report improved secondary prevention outcomes, including increased exercise capacity and physical activity levels. These trials provided at least one contact session per fortnight^52^ to deliver feedback to support lifestyle modification and improve secondary prevention outcomes. Session delivery occurred through different telehealth modalities, including videoconferencing,^61^ telephone, and instant messaging^51^ platforms. Maintaining repetitive contact between participants and clinicians appeared to increase programmes’ perceived usefulness, leading to increased participation and acceptability in telehealth-delivered CR interventions.

The repetitive contact between participants and clinicians was vital for improving participation rates and self-management. Repetitive contact facilitated intervention engagement and self-management skills provision to enhance participants’ satisfaction rates, participation, and perceived usefulness. At the same time, repetitive contact was also critical to reducing anxiety and depression among participants in telehealth-delivered CR care. Similarly to early engagement, repetitive contact could also benefit participation and secondary prevention outcomes when provided during CR Phase 1.

##### Theory 2: personalize interventions and develop shared goals

Personalized telehealth-delivered CR interventions were more likely to report improved secondary prevention outcomes and increased participation and satisfaction by including strategies adjusted to participants’ needs and goals. These strategies included providing feedback embedded in behavioural change theories, ensuring the intervention is available in multiple modalities to suit the participant’s needs, and providing bi-directional opportunities^47,59^ for contact. Consequently, these personalization strategies included in telehealth-delivered CR interventions were reported to improve acceptability and feasibility of goals, resulting in increased motivation and satisfaction rates among patients, which potentially led to increased secondary prevention outcomes. For example, as Sankaran *et al.*^39^ reported, a personalized intervention (including personalized feedback delivered by the clinician) could increase participants’ comprehension of their own progress and ultimately achieve physical activity goals effectively. Neubeck *et al.* also recommended a participant-specific approach since actively engaging patients in decision-making may have increased participation rates. Additionally, bi-directional interaction can allow greater personalization due to the increased opportunity for feedback and support.^49^ On the other hand, lack of personalization may result in loss of interest in the intervention and contribute to null effects on secondary prevention outcomes. Most of our included studies were more likely to report that telehealth-delivered CR interventions could improve results when a personalized approach is included within service delivery.

Engaging participants in decision-making appeared to increase participation rates. In order to develop shared goals, interventions included behaviour change theories, such as goal setting and motivational interviewing. This feature required repetitive contact between participants and clinicians, where feedback provision was possible. Feedback aimed to increase self-efficacy by promoting competence among participants to support the adoption of lifestyle changes, which could lead to improved exercise capacity.^62^ Additionally, established bi-directional communication with repeated contact sessions (e.g. telecoaching) supported patients to achieve pre-defined goals in telehealth-delivered CR interventions. As a result, increased self-efficacy contributed to boosting confidence and adherence to the interventions.^62,63^ As reported by Kraal *et al.*,^64^ by providing feedback through telecoaching, participants were empowered to execute an exercise routine at home independently and, at the same time, achieve the outlined goals from the exercise prescription.^64,65^

Feedback timeliness did not appear to influence goal achievement. Asynchronous feedback,^59^ such as in chat platforms and texting, worked as a medium to provide emotional support and targeted education that supported lifestyle changes. Real-time feedback provision increased participation and improved secondary outcomes by maintaining participants’ confidence in the intervention.^19,41,55^ Authors primarily used RPM technologies to provide real-time support about exercise execution and reduce exercise-related anxiety.^38^

##### Theory 3: ensure interventions are usable, accessible, and supported

Technological interventions need to be easy to use and adjusted to the participants’ digital health literacy. Across the telehealth interventions, the authors highlighted the need to ensure adequate internet access and technological provisions for participants. For example, Guiraud *et al.*^59^ relied on the widespread knowledge of telephone use to provide counselling sessions that included support and feedback to increase physical activity levels. Similarly, Leemrijse *et al.*,^44^ who used telephone calls, and Dorje *et al.*,^55^ who used a smartphone application, highlighted the impact of telehealth-delivered CR interventions on improving secondary outcomes when telehealth delivery CR interventions are accessible and easy to use by participants. Consequently, accessing these types of interventions yielded additional benefits from the programmes, such as awareness and self-efficacy, vital for goal achievement on secondary prevention outcomes, participation, and participants’ satisfaction.

Easy-to-use technologies were required to effectively establish bi-directional communication between participants and clinicians through different platforms such as text messaging,^66^ RPM,^38,67^ and web platforms.^38,67,68^ These interventions should include user-friendly features to prevent participants’ frustration and delays in feedback delivery. Familiarization and demonstration of the technologies was important to increase user confidence. For some (e.g. Kraal *et al.*^64^), this involved supervised training with instructions on how to use devices and upload data. Many studies reported high education levels of their participants^69^ and exclusion of participants that did not have access to the internet or computer at home.^64^ Therefore, supporting digital health literacy may be even more important in the general population. Skobel *et al.*^56^ and Kayser *et al.*^60^ developed systems that resulted in poor usability and connectivity of the technological device and negatively influenced participation outcomes. Moreover, strict safety protocols preventing app use^57^ and lack of technical support^43^ also impact the ease of use, limiting participation.

##### Theory 4: measure and monitor exercise

Measuring exercise appears to contribute to maintaining participants’ motivation to continue with the intervention, optimize satisfaction rates, and improve secondary prevention outcomes. Across telehealth interventions, authors agreed that measuring exercise achievements and improving physical activity levels (objectively or self-reported) motivated participants to achieve pre-set CR goals. They increased the perceived usefulness of the intervention.^55,59,64,70^ Remote patient monitoring technologies (i.e. wearable devices and/or smartphones that collected biometric information) were the preferred modality to measure and monitor exercise. Synchronous and asynchronous exercise measurements both benefited exercise outcomes^47,71^ and supported the adoption of physical activity habits.^51^ Similar to our realist Theory 2, feedback embedded in behavioural change theories, such as motivational interviewing and goal setting, was the proposed mechanism.^65,70,71^ In this sense, measuring and monitoring exercises required accessible and easy-to-use telehealth-delivered CR technologies (Theory 3) to achieve effective care delivery.^41^ However, a lack of exercise measurement and monitoring could reduce participation.^69^

#### Recommendations for future telehealth use within cardiac rehabilitation

Recommendations for optimizing outcomes for patients participating in telehealth-delivered CR include the following: having low-cost technology or providing devices, offering a variety of platforms and modalities of care, tailoring exercise prescriptions, objectively measuring exercise, and having regular two-way contact between the patient and provider. Patients should also be engaged early with the intervention (e.g. before being discharged from the hospital) and should have a choice on their preferred modality of care.

## Discussion

We found personalized approaches in telehealth-delivered CR interventions with repetitive bi-directional interaction between the health provider and participants are key success factors for improving secondary prevention outcomes (i.e. BMI, lipid profile, smoking cessation, and physical activity), exercise capacity, participation, and satisfaction in participants. Repetitive interaction that leverages behavioural change theories promoted the adoption of lifestyles favouring cardiovascular health. Participants need to be engaged early and often and access telehealth-delivered care through accessible, usable, and supported interventions.

To the best of our knowledge, this is the first review to analyse key features of telehealth-delivered CR using a realist synthesis approach. Gass *et al.*^72^ narratively reviewed papers across preventative cardiology populations to investigate the impact of telemedicine design features on adherence. Further, Ramachandran *et al.*^31^ have previously reported equivalent effects on secondary prevention outcomes when telehealth-delivered CR was compared with facility-based interventions and increased effectiveness compared with usual care.

Aligning to Theory 1, Gass *et al.*^72^ also highlighted the need for recurrent personal contact (either in-person or via phone or video) to promote adherence and participation in telehealth-delivered programmes. The broader (non-telehealth specific) literature also supports regular clinician–patient engagement as a key factor in promoting participation,^73^ with evidence to show earlier enrollment in CR after a patient is discharged leads to improved attendance.^74^ This appears to be more successful when delivered by either nurses or allied health professionals.^14^ Potentially, this early engagement facilitates positive clinician–participant relationships and rapport building previously described in the literature to facilitate successful CR outcomes.^75^

Similar to our results (Theory 2), Ramachandran *et al.*^31^ acknowledged the effect of providing personalized interventions that target self-efficacy and are underpinned by behavioural change theories. Personalization and bi-directional feedback have previously been reported as crucial factors influencing the effectiveness of home-based CR programmes.^31^ Here, we provide additional evidence to be considered regarding the importance of targeted feedback in regard to exercise monitoring (Theory 4)—this is an area where RPM devices can really enhance the information received by both the clinician and patient.

To increase telehealth-delivered CR implementation effectiveness, Batalik *et al.*^76^ recommended a comprehensive approach including increasing health e-literacy support and social support in the intervention. Our findings support the necessity to provide support for technology adoption and to increase trust and acceptability among participants toward the intervention (Theory 3). The best way to do this requires further investigation. Moreover, the Ramachandran *et al.*^31^ review added a recommendation to consider the place of data privacy on participant willingness to engage with telehealth-delivered CR programmes. Although data privacy is an important consideration, this did not appear among perceived factors affecting intervention outcomes in our review. To examine this aspect, specific assessments of acceptability may be needed.

### Strengths and limitations

Our review identified limitations in the design and reporting of the included studies. In most studies, blinding of participants was impossible because of the studies’ settings (telehealth-delivered interventions vs. facility-based or usual care). Included study populations lacked diversity with most studies excluding people with disability, sensory impairments, cognitive impairments, and English as a second language. Included population groups were largely from metropolitan areas, under 75 years old, spoke English, and had good digital literacy. Additionally, all studies had a higher proportion of males to females (the majority of studies reported >70% of included participants were male). Research has shown an underrepresentation of women in cardiovascular trials relative to proportion of women with cardiovascular disease. Consequently, our findings may not adequately represent the needs of women. Authors’ reports about clinicians’ and other care providers’ contributions to the intervention were limited. In this sense, there needs to be improved quality of reporting of clinical trials by promoting clinical trial reporting to adhere to current guidelines (e.g. CONSORT).

This study has many strengths. We included a wide range of databases in our search, studies from different parts of the world, and large sample sizes that increase this review’s external validity. We also explored multiple technologies with varied familiarity and complexity (e.g. telephone or short-text messages and virtual reality or remote participant monitoring). While interventions included features described in the four realist theories, these seemed effective irrespective of their intervention delivery technologies.

### Future directions

As participants’ acceptability of programmes had an impact on clinical outcomes, pragmatic RCTs should be considered to enable participant choice in their model of care. Additionally, large-scale, ‘real-world’ effectiveness studies are needed to provide evidence of how telehealth-delivered CR programmes perform when implemented in complex clinical settings. We note some clinical trials currently underway (e.g. the RESTORE trial being conducted in Poland^77^ and the LeIKD trial in Germany^78,79^) may provide new lessons in this area. Such studies need to incorporate data on patient choice to use or not use the programme and perception and beliefs of telehealth-delivered CR, and this will support learnings in the patient groups most likely to benefit from telehealth options. Studies should also properly report clinician involvement in the programme delivery as this has implications for workforce use and planning. Our review did not find relevant information about how best to engage clinicians in telehealth-delivered CR interventions. We also agree with other recently articulated CR research priorities including that future work must address how telehealth-delivered CR can be adapted to support more diverse populations.^80^

## Conclusions

Our realist review provides a practical resource that contributes to telehealth-delivered CR service implementation. A personalized approach with opportunities for early and regular bi-directional interaction and inclusion of exercise monitoring appears to be key ingredients in engaging and effective programmes. Additionally, it cannot be understated the importance of ensuring these programmes are easy to use with appropriate technical supports.

## Supplementary Material

ztad080_Supplementary_Data

## Data Availability

No new data were generated or analysed in support of this research.

## Lead author biography

Victor M. Gallegos-Rejas, MD, MPH, by training, is a medical doctor and started his professional career in rehabilitation medicine. He completed a master’s degree in public health at The University of Queensland (UQ). Before starting his studies at UQ, Victor worked as a sports medical specialist in his natal Peru, increasing access to medical care for high-performance athletes. In his PhD, Victor aims to facilitate the uptake of telehealth through the equity lens. In his project, Victor investigates equity of access to telehealth, evaluates strategies to reduce the digital divide, and proposes co-designed solutions to promote equitable access to telehealth.
