# Supplementary material for: Key features in telehealth-delivered cardiac rehabilitation required to optimize cardiovascular health in coronary heart disease: a systematic review and realist synthesis
Source: Eur Heart J Digit Health. 2024 Jan 5;5(3):208–18. doi: 10.1093/ehjdh/ztad080 (PMC11104477; doi:10.1093/ehjdh/ztad080)
Supplement: ztad080_Supplementary_Data [file ztad080_supplementary_data.zip › Appendices.docx]

# Appendices

## Tables

Supplementary Table 1. PubMed Search Terms

| Database | Search string |
| --- | --- |
| PubMed / Cochrane Database | ((((((((((((((((((((((((((((("Angioplasty"[Mesh]) OR ("angioplasties"[Title/Abstract])) OR (Angioplasty[Title/Abstract])) OR ("chronic coronary syndrome"[Title/Abstract])) OR ("acute coronary syndrome"[Title/Abstract])) OR ("angina"[Title/Abstract])) OR ("Myocardial Ischaemia"[Title/Abstract])) OR ("Myocardial Ischemia"[Title/Abstract])) OR ("heart disease"[Title/Abstract])) OR ("heart diseases"[Title/Abstract])) OR ("coronary heart disease"[Title/Abstract])) OR ("ischemic heart disease"[Title/Abstract])) OR ("ischaemic heart disease"[Title/Abstract])) OR ("percutaneous coronary intervention"[Title/Abstract])) OR ("coronary artery bypass"[Title/Abstract])) OR ("myocardial infarction"[Title/Abstract])) OR ("heart failure"[Title/Abstract])) OR ("coronary disease"[Title/Abstract])) OR ("coronary artery disease"[Title/Abstract])) OR ("Acute Coronary Syndrome"[Mesh])) OR ("Coronary Artery Bypass"[Mesh])) OR ("Myocardial Infarction"[Mesh])) OR ("Heart Failure"[Mesh])) OR ("Myocardial Ischemia"[Mesh])) OR ("Coronary Disease"[Mesh])) OR ("Percutaneous Coronary Intervention"[Mesh])) OR ("Myocardial Revascularization"[Mesh])) OR ("Angina Pectoris"[Mesh])) AND ((((((((((((((((((((((((telehealth[Title/Abstract]) OR ("Telemedicine"[MeSH Terms])) OR ("Telemedicine"[Title/Abstract])) OR ("Telerehabilitation"[MeSH Terms])) OR ("Telerehabilitation"[Title/Abstract])) OR ("internet"[MeSH Terms])) OR (internet[Title/Abstract])) OR (skype[Title/Abstract])) OR (sms[Title/Abstract])) OR ("smartphone"[MeSH Terms])) OR ("smartphone"[Title/Abstract])) OR ("mobile phone"[Title/Abstract])) OR (teleconsultation*[Title/Abstract])) OR ("videoconferencing"[MeSH Terms])) OR ("videoconferencing"[Title/Abstract])) OR (videoconference[Title/Abstract])) OR ("telemetry"[MeSH Terms])) OR ("telemetry"[Title/Abstract])) OR ("telemetries"[Title/Abstract])) OR ("Remote Consultation"[Mesh])) OR ("Cell Phone"[MeSH Terms])) OR ("cell phone"[Title/Abstract])) OR (telemonitor*[Title/Abstract])) OR ("Cardiac Rehabilitation"[Mesh]))) AND (((((("Randomized Controlled Trial"[Publication Type]) OR (Randomized Controlled Trials as Topic[MeSH Terms])) OR ("clinical trials as topic"[MeSH Terms])) OR ("clinical trial*"[All Fields])) OR ("random*"[Title/Abstract])) OR ("RCT"[Title/Abstract])) |

Supplementary Table 2. Study description

|  | Lead Author (Year), Country of Origin | Sample size (n) | Age (years) | Modality of telehealth-delivered CR care | Description of the intervention | Duration of the intervention | Outcomes measured |
| --- | --- | --- | --- | --- | --- | --- | --- |
| Independent Studies | | | | | | | |
| 1 | Lear (2015), Canada ^66^. | 78 | - IG: 58.4 - CG: 61.7 | RPM  Web-based application  Phone | - 1 training session - 3 nurse chat sessions over a 12-week period - 3 exercise specialist sessions over a 12-week period - 3 dietitian sessions over a 12-week period - 1 education session per week - 1 group session over a 4-week period | 12 weeks | - Lipid profile - Blood pressure - Smoking cessation - Changes in body composition - Self-reported PA - Exercise capacity |
| 2 | Lee (2013), Korea ^45^. | 55 | - IG: 54 - CG: 57 | RPM | - 4-5 exercise sessions per week, each session 50 mins. - 1 counselling session per week, including education and tailoring. | 12 weeks | - Blood pressure - Health-related Quality of Life (including anxiety) - Exercise capacity |
| 3 | Bravo-Escobar (2017), Spain ^35^. | 28 | - IG 65.5 - CG: 55.6 | RPM  Built-in Application | - 3 exercise sessions per week, hybrid delivery. - 1 education session per week, facility-based. - 1 group session per week | 8 weeks | - Blood pressure - Changes in body composition - Exercise capacity |
| 4 | Noites (2017), Portugal ^52^. | 32 | - IG: 62.5 - CG: 59.5 | RPM  Phone call  Text-messaging | - 4 Educational sessions over an 8-week period - Tailored exercise programme - 1 remote supervision session per week - 1 counselling session per fortnight | 8 weeks | - Resting blood pressure - Changes in body composition - Objective daily PA levels - Exercise capacity |
| 5 | Guiraud (2012), France ^59^. | 29 | - IG: 54.5 - CG: 62.9 | Phone + RPM | - Exercise tracking - 1 counselling session per fortnight (included education) | 8 weeks | - Objective PA - Energy expenditure |
| 6 | Fang (2019), China ^47^. | 80 | - IG: 61 - CG: 60 | RPM, built-in application, web-based application, Phone | - 1 educational booklet - 3 or more synchronous monitored exercise sessions per week - 1 counselling session per week - RPM App | 6 weeks | - Smoking cessation - Health-related quality of life - Depression - Exercise capacity |
| 7 | Devi (2014), United Kingdom ^48^. | 94 | - IG: 48 | Web-based application | - 3-4 log-in to a web-based application including tailored goals focused on exercise and education. - Feedback and support available online. - 1 chat session per week. | 6 weeks | - Blood pressure - Changes in Body composition - Anxiety and depression - Health-related quality of life - Objective PA |
| Fit@Home Study | | | | | | | |
| 8 | Kraal (2017), The Netherlands ^75^. | 90 | - IG: 54 - CG: 57 | Web-based application | - 3 supervised technology training sessions - 2 or more monitored exercise sessions per week - 1 Phone Feedback Session per week - Access to web-based application for additional support | 12 weeks | - Health-related quality of life - Psychosocial status - Objective PA - Exercise capacity - Patient satisfaction |
| 9 | Kraal (2014), The Netherlands ^63^. | 50 | - IG: 54 - CG: 57 | Web-based application |  | 12 weeks |  |
| CAP-CR Study | | | | | | | |
| 10 | Varnfield (2014), Australia ^43^. | 120 | - IG: 54.9 - CG: 56.2 | Designed Application | - 2 supervised exercise session per week - 1 education session per week - 1 tele coaching session per week - Access to educational platform available via text-messaging and web portal. | 6 weeks | - Uptake, participation, and adherence to intervention - Objective PA - Lipid profile - Changes in body composition - Health-related quality of life - Depression and anxiety - Exercise capacity |
| CardioFit Study | | | | | | | |
| 11 | Reid (2012), Canada ^67^. | 223 | - IG: 56.7 - CG: 56 | Web-based application | - Early engagement at hospital. - Exercise tracking - 5 online education sessions over a 24-week period - Access to web-based application | 24 weeks | - Body composition - Objective PA - Health-related PA. |
| Text4Heart Study | | | | | | | |
| 12 | Pfaeffli Dale (2015), New Zealand ^49^. | 123 | - IG: 59.0 - CG: 59.9 | Phone + text messaging | - Access to website for educational material. - 5-7 short text messages per week | 24 weeks | - Body composition - Blood pressure - Lipid profile - Smoking cessation - Self-reported PA |
| HeartHab Study | | | | | | | |
| 13 | Sankaran (2019), Belgium ^39^. | 32 | - IG: 60.9 - CG: 60.9 | Built-in Application | - Access to patient-tailored app with educational content. - Hybrid model of care | 8 weeks | - Changes in body composition - Blood pressure - Lipid profile - Glucose - Health-related quality of life (including anxiety) - Self-reported PA - Exercise capacity |
| HeartCycle Study | | | | | | | |
| 14 | Salvi (2018), Spain ^57^. | 118 | - IG: 59 - CG: 59 | Designed Application | - Access to application for educational support - 2-3 tailored supervised exercise sessions (included feedback) per week, sessions varied from 10 to 60 minutes. - Tailored text message delivery | 24 weeks | - Lipid profile - User acceptance and perceived usefulness - Health-related quality of life - Objective PA - Exercise capacity |
| 15 | Skobel (2017), Germany ^56^. | 132 | - IG: 59 | Designed Application |  | 24 Weeks |  |
| REMOTE-CR Study | | | | | | | |
| 16 | Rawstorn (2018), Australia ^41^. | 162 | - IG: 61.9 | RPM | - 3 supervised tailored exercise sessions per week, each session between 30 to 60 minutes (included real time feedback) - Tele coaching sessions - Access to monitoring app. - Short text message delivery | 12 weeks | - Body composition - Blood pressure - Lipid profile - Glucose - Health-related quality of life - Acceptability - Exercise capacity |
| 17 | Maddison (2019), New Zealand ^19^. | 162 | - IG: 61.9 | RPM |  | 12 weeks |  |
| EU-CaRE Study | | | | | | | |
| 18 | Snoek (2020), The Netherlands ^50^. | 179 | - Mean: 73.6 | Built-in Application | - 1 counselling session per week over 2-month period - 1 counselling session per month over a 4-month period - 5 tracked exercise session per week, each session 30 minutes - Access to mobile application for exercise tracking | 24 weeks | - Body composition - Health-related quality of life - Anxiety and depression - Self-reported PA - Exercise capacity |
| 19 | Snoek (2019), The Netherlands ^50^. | 122 | - Mean: 73.6 | Built-in Application |  | 24 weeks + 12 weeks of follow-up |  |
| Kinect Study | | | | | | | |
| 20 | Vieira (2017), Portugal ^37^. | 46 | - IG: 55 - CG: 59 | Virtual Reality | - 3 monitored exercise sessions per week - Education - Access to Virtual Reality platform - Real time feedback | 24 weeks | - Body composition - Health-related quality of life - Lipid profile - Objective PA |
| 21 | Vieira (2018), Portugal ^61^. | 46 | - IG: 55 - CG: 59 | Virtual Reality |  | 24 weeks |  |
| TEXT-ME Study | | | | | | | |
| 22 | Thakkar (2016), Australia ^36^. | 710 | - IG: Not reported - CG: Not reported | Text messaging | - Hybrid model of care with text message support - 1 text message per day, includes educational content | 24 weeks | - Self-reported PA |
| Telerehab-III Study | | | | | | | |
| 23 | Frederix (2015), Belgium ^65^. | 80 | - IC: 81 - CG: 85 | RPM | - Hybrid model of care - 1 text message per day, including tele coaching and feedback support - 1 text message per day, including education support. - Monitored PA behaviour | 18 weeks | - Glucose - Lipid profile - Health-related quality of life - Objective PA - Self-reported PA - Exercise capacity |
| 24 | Frederix (2015), Belgium ^46^. | 140 | - IC: 61 - CG: 61 | RPM |  | 24 weeks |  |
| 25 | Frederix (2016), Belgium ^65^. | 140 | - IC: 61 - CG: 61 | RPM |  | 24 weeks |  |
| HEART mHealth Study | | | | | | | |
| 26 | Maddison (2015), New Zealand ^76^. | 171 | - Mean: 60.2 | Phone + Text | - 5 or more exercise sessions per week, each session of 30 minutes or more. - 1 or more logins to website for educational support - 3-5 text messages per week, included behavioural change strategies. | 24 weeks | - Health-related quality of life - Self-efficacy - Self-reported PA - Exercise capacity |
| 27 | Maddison (2014), New Zealand ^62^. | 171 | - Mean: 60.2 | Phone + Text |  | 24 weeks |  |
| 28 | Pfaeffli Dale (2015), New Zealand ^64^. | 171 | - IG: 61.38 - CG: 63.7 | Text messaging  Web-based application |  | 24 Weeks |  |
| Heartcoach Study | | | | | | | |
| 29 | Leemrijse (2016), The Netherlands ^44^. | 371 | - IG: 61.0 - CG: 60 | Phone Call | - Hybrid model of care - 1 coaching session per month, including behavioural change strategies | 24 weeks | - Body composition - Blood pressure - Lipid profile - Glucose - Self-efficacy - Anxiety and depression - Health-related quality of life - Self-reported PA |
| TRiCH Study | | | | | | | |
| 30 | Avila (2018), Belgium ^54^. | 90 | - IG: 61.2 | Designed Application | - 6-7 individualised exercise sessions per week - 1 feedback session per week, using phone or email. - Access to application for exercise tracking and educational support | 12 weeks | - Body composition - Lipid profile - Homeostasis assessment model index - Objective PA - Exercise capacity |
| 31 | Avila (2020), Belgium ^77^ | 90 | - IG: 62.6 | Designed Application |  | 12 weeks |  |
| TAIVE en m@rche Study | | | | | | | |
| 32 | Kayser (2019), Canada ^60^ | 60 | - IG: 59.2 - CG: 58.6 | Web-based application | - 4 introductory sessions to platform - Access to a website with educational support. - Individualised text message support | 12 weeks | - Objective PA - Self-reported PA |
| CHOICE Study | | | | | | | |
| 33 | Neubeck (2011), Australia ^53^ | 142 | - Mean: 64 | Phone Call | - 2 group-based exercise session per week, each session of 60 minutes - 1 educational session per week, each session of 2 hours. - Multiple telephone support session. | 12 weeks | - Body composition - Lipid profile - Depression - Smoking cessation - Self-reported PA |
| RENATA Study | | | | | | | |
| 34 | Duan (2018), Hong Kong ^58^ | 114 | - IG: 45.8 - CG: 51.57 | Web-based application | - 1 login to website per week for educational support - Text message delivery for support - 1 tele counselling session per week | 8 weeks | - Body composition - Self-reported PA - Self-efficacy - Quality of life - Depression |
| SMART-CR/SP Study | | | | | | | |
| 35 | Dorje (2019), China ^55^. | 312 | - IG: - CG: | Designed application | - Access to instant message for service delivery and support - Automatic data transmission of alerts and messages. - 5 or more tracked exercise sessions, each session of 30 minutes. | 24 weeks | - Body composition - Lipid profile - Blood pressure - Smoking cessation - Objective PA - Exercise capacity |
| 36 | Yudi (2020), Australia ^51^. | 206 | - IG: 56.8 - CG: 56.2 | Designed application |  | 8 weeks |  |
| MEMRS-CRS Study | | | | | | | |
| 37 | Song (2020), China ^38^. | 106 | - IG: 54.17 - CG: 54.83 | RPM | - Monitored exercise sessions - 1 tele counselling session per week - 1 feedback session per week - Access to instant messaging platform | 24 weeks | - Body composition - Lipid profile - Glucose - Blood pressure - Exercise capacity |

CG: Control group; IG: Intervention group; PA: Physical activity; RPM: remote patient monitoring

Supplementary Table 3. Factors for optimised outcomes in telehealth-delivered cardiac rehabilitation

| **Factors for optimised outcomes** | **Reduced cardiovascular risk factors** | **Improved exercise capacity** | **Improved participation** | **Increased satisfaction** |
| --- | --- | --- | --- | --- |
| **Technology – related factors** |  |  |  |  |
| **Low-cost technology** (delivered via text messaging or phone call.) | Reid (2012)^67^ , Dale (2015)^49^, Thakkar (2016)^36^, Leemrijse (2016)^44^ |  | Lear (2015)^66^, Guiraud (2012)^59^, Neubeck (2011)^53^, SMART-CR/SP: Dorje (2019)^55^, Yudi (2020)^51^ |  |
| **Programme reached in multiple platforms** (e.g., website and mobile application, mobile application and booklet, etc.) | Lear (2015)^66^, Dale (2015)^49^, Fang (2019)^47^, REMOTE-CR: Rawstorn (2018)^41^, Maddison (2019)^19^, Duan (2018)^58^, SMART-CR/SP: Dorje (2019)^55^, Yudi (2020)^51^ | Salvi (2018)^57^, REMOTE-CR: Rawstorn (2018)^41^, Maddison (2019)^19^, SMART-CR/SP: Dorje (2019)^55^, Yudi (2020)^51^ | Rawstorn (2016)^24^, Lear (2015)^66^, Neubeck (2011)^53^, Duan (2018)^58^, SMART-CR/SP: Dorje (2019)^55^, Yudi (2020)^51^ | Rawstorn (2016)^24^, REMOTE-CR: Rawstorn (2018)^41^, Maddison (2019)^19^ |
| **Easy-to-use technology** | Guiraud (2012)^59^, Dale (2015)^49^, Leemrijse (2016)^44^ |  | Thakkar (2016)^36^, Neubeck (2011)^53^, SMART-CR/SP: Dorje (2019)^55^, Yudi (2020)^51^ | Rawstorn (2018)^41^, Maddison (2019)^19^, Frederix (2016)^65^, SMART-CR/SP: Dorje (2019)^55^, Yudi (2020)^51^ |
| **Using a hybrid model of care** (included home-based exercise and facility-based education or viceversa) | Leemrijse (2016)^44^ | Varnfield (2014)^43^ | Noites (2017)^52^, Kraal (2014)^63^, Kraal (2017)^75^ | Fit@Home: Kraal (2014)^63^, Kraal (2017)^75^ |
| **Gamification of the intervention** | SMART-CR/SP: Dorje (2019)^55^ |  | SMART-CR/SP: Dorje (2019)^55^ | TRiCH: Avila (2018)^54^, Avila (2020)^77^ |
| **Provision of technological resources** (including smartphones or wearable sensors for RPM) | EU-CaRE: Snoek (2019)^50^, Snoek (2020)^50^, TRiCH: Avila (2018)^54^, Fang (2019)^47^ | Telerehab-III: Frederix (2015)^46^, Frederix (2016)^65^, Frederix (2015)^78^, Fang (2019)^47^ | TRiCH: Avila (2018)^54^ | Rawstorn (2018)^41^ |
| **Factors related to component of care** |  |  |  |  |
| **Multiple modalities of care** (e.g. tailored education, comprehensive CR programs) | Dale (2015)^49^, Guiraud (2012)^59^, Fang (2019)^47^, Kinect: Viera (2017), Vieira (2018)^61^, Telerehab-III: Frederix (2015)^46^, Frederix (2016)^65^, Frederix (2015)^78^, SMART-CR/SP: Dorje (2019)^55^, Yudi (2020)^51^ | Salvi (2018)^57^, Varnfield (2014)^43^, Telerehab-III: Frederix (2015)^46^, Frederix (2016)^65^, Frederix (2015)^78^ | Lear (2015)^66^, Guiraud (2012)^59^, SMART-CR/SP: Dorje (2019)^55^, Yudi (2020)^51^ |  |
| **Tailored exercise prescription** (or physical activity plan) | Reid (2012)^67^, REMOTE-CR: Rawstorn (2018)^41^, Maddison (2019)^19^, TRiCH: Avila (2018)^54^ | Fang (2019)^47^, Lee (2013)^45^, Sankaran (2019)^39^, Telerehab-III: Frederix (2015)^46^, Frederix (2016)^65^, Frederix (2015)^78^, TRiCH: Avila (2018)^54^ | Sankaran (2019)^39^, TRiCH: Avila (2018)^54^ | Fit@Home: Kraal (2014)^63^, Kraal (2017)^75^ |
| **Exercise tracking** (e.g. using objectives measures for physical activity measurement) | Lee (2013)^45^, Guiraud (2012)^59^, Fang (2019)^47^, Devi (2014)^48^, Reid (2012)^67^, Snoek (2020)^50^, Telerehab-III: Frederix (2015)^46^, Frederix (2016)^65^, Frederix (2015)^78^, TRiCH: Avila (2018)^54^, Avila (2020)^77^, SMART-CR/SP: Dorje (2019)^55^, Yudi (2020)^51^, Song (2020)^38^ | Varnfield (2014)^43^, Salvi (2018)^57^, Noites (2017)^52^, Fang (2019)^47^, TRiCH: Avila (2018)^54^, SMART-CR/SP: Dorje (2019)^55^, Yudi (2020)^51^ | Noites (2017)^52^, SMART-CR/SP: Dorje (2019)^55^, Yudi (2020)^51^ | Fit@Home: Kraal (2014)^63^, Kraal (2017)^75^, Rawstorn (2018)^41^ |
| **Targeted self-efficacy** (including health-literacy and/or e-literacy) | Fang (2019)^47^, Devi (2014)^48^, Reid (2012)^67^, Dale (2015)^49^, HEART mHealth: Maddison (2015)^76^, Leemrijse (2016)^44^, Duan (2018)^58^ | Lee (2013)^45^ | TRiCH: Avila (2018)^54^, Avila (2020)^77^, Neubeck (2011)^53^, Duan (2018)^58^, SMART-CR/SP: Dorje (2019)^55^, Yudi (2020)^51^ | Fit@Home: Kraal (2014)^63^, Kraal (2017)^75^, HEART mHealth: Maddison (2015)^76^, Pfaeffli Dale (2015)^64^, Maddison (2014)^62^ |
| **Factors related to the clinician-patient relationship** |  |  |  |  |
| **Two-way communication between provider and patient** | Sankaran (2019)^39^, Guiraud (2012)^59^, Fang (2019)^47^, Devi (2014)^48^, EU-CaRE: Snoek (2019)^50^, Snoek (2020)^50^, Frederix (2015)^46,78^, Leemrijse (2016)^44^, TRiCH: Avila (2020)^77^, Song (2020)^38^, Lack of: Thakkar (2016)^36^ | Sankaran (2019)^39^, TRiCH: Avila (2020)^77^ | Sankaran (2019)^39^, Guiraud (2012)^59^, Fit@Home: Kraal (2014)^63^, Kraal (2017)^75^, Neubeck (2011)^53^, Song (2020)^38^ | Fit@Home: Kraal (2014)^63^, Kraal (2017)^75^ |
| **Feedback embedded in behavioural change theories** | Sankaran (2019)^39^, Salvi (2018)^57^, Guiraud (2012)^59^, Reid (2012), Dale (2015)^49^, EU-CaRE: Snoek (2019)^50^, Snoek (2020)^50^, Kinect: Viera (2017)^37^, Vieira (2018)^61^, Telerehab-III: Frederix (2015)^46^, Frederix (2016)^65^, Frederix (2015)^78^, HEART mHealth: Maddison (2015)^76^, Pfaeffli Dale (2015)^49^, Leemrijse (2016)^44^, TRiCH: Avila (2018)^54^, SMART-CR/SP: Dorje (2019)^55^, Yudi (2020)^51^ | Snoek (2020)^50^, Telerehab-III: Frederix (2015)^46^, Frederix (2016)^65^, Frederix (2015)^78^, TRiCH: Avila (2018)^54^ | Guiraud (2012)^59^, Neubeck (2011)^53^, Duan (2018)^58^, SMART-CR/SP: Dorje (2019)^55^, Yudi (2020)^51^ | Fit@Home: Kraal (2014)^63^, Kraal (2017)^75^, REMOTE-CR: Rawstorn (2018)^41^, Maddison (2019)^19^, SMART-CR/SP: Dorje (2019)^55^, Yudi (2020)^51^ |
| **Regular contact with patients** (provided contact at least weekly) | Noites (2017), Guiraud (2012)^59^, Fang (2019)^47^, Dale (2015)^49^, Kinect: Vieira (2017)^37^, Thakkar (2016)^36^, Telerehab-III: Frederix (2015)^46^, Frederix (2016)^65^, Frederix (2015)^78^, Leemrijse (2016)^44^, SMART-CR/SP: Dorje (2019)^55^, Yudi (2020)^51^ | Varnfield (2014)^43^, Noites (2017), Snoek (2020)^50^, Telerehab-III: Frederix (2015)^46^, Frederix (2016)^65^, Frederix (2015)^78^ | Noites (2017)^52^, Guiraud (2012)^59^, Neubeck (2011)^53^, Duan (2018)^58^, SMART-CR/SP: Dorje (2019)^55^, Yudi (2020)^51^ | Kraal (2014)^63^, Kraal (2017)^63^, Rawstorn (2018)^41^, HEART mHealth: Maddison (2015)^76^, Pfaeffli Dale (2015)^49,64^, Maddison (2014)^62^ |
| **Patient provided with choice on preferred modality of care** | EU-CaRE: Snoek (2019)^50^, Snoek (2020)^50^ |  |  |  |
| **Patients' previous engagement in physical activity** | Guiraud (2012)^59^, Thakkar (2016)^36^, Telerehab-III: Frederix (2015)^46^, Frederix (2016)^65^, Frederix (2015)^78^, Duan (2018)^58^, Song (2020)^38^ | Noites (2017)^52^, Telerehab-III: Frederix (2015)^46^, Frederix (2016)^65^, Frederix (2015)^78^ | Thakkar (2016)^36^, Duan (2018)^58^, Song (2020)^38^ |  |
| **Research team engaged with clinicians** |  | Maddison (2019)^19^, Fang (2019)^47^ | Fit@Home: Kraal (2014)^63^, Kraal (2017)^75^ |  |
| **Early engagement with the intervention** (including during hospital admission) | Lee (2013), SMART-CR/SP: Dorje (2019)^55^, Yudi (2020)^51^ |  | SMART-CR/SP: Dorje (2019)^55^, Yudi (2020)^51^ | Lee (2013)^45^ |

Supplementary Table 4. Factors for reduced outcomes

| **Factors for reduced outcomes** | **No difference in cardiovascular risk factors** | **No difference in exercise capacity** | **Reduced or unmodified participation** | **Reduced satisfaction** |
| --- | --- | --- | --- | --- |
| **Technology-related factors** |  |  |  |  |
| **Poor usability of the technological device** (including strict safety mechanisms) | Skobel (2017)^56^, Kayser(2019)^60^ |  | Varnfield (2014)^43^, HeartCycle: Salvi (2018)^57^, Skobel (2017)^56^ | HeartCycle: Salvi (2018)^57^, Skobel (2017)^56^, Kayser(2019)^60^, Song (2020)^38^ |
| **Poor connectivity** (i.e. reduced ability to connect to the device or platform) | Skobel (2017)^56^, Kayser(2019)^60^ |  | HeartCycle: Salvi (2018)^57^, Skobel (2017)^56^ |  |
| **Using a hybrid model of care** (included home-based exercise and facility-based education or viceversa) | Bravo-Escobar (2017)^35^, Fit@Home: Kraal (2014)^63^, Kraal (2017)^75^, Kinect: Viera (2017)^37^, Vieira (2018)^61^ | Bravo-Escobar (2017)^35^, Fit@Home: Kraal (2014)^63^, Kraal (2017)^75^ | Kinect: Viera (2017)^37^, Vieira (2018)^61^, Leemrijse (2016)^79^ |  |
| **Factors related to component of care** |  |  |  |  |
| **Multiple component care** (e.g. tailored education) | Bravo-Escobar (2017)^35^, Fit@Home: Kraal (2014)^63^, Kraal (2017)^75^ | Bravo-Escobar (2017)^35^, Fit@Home: Kraal (2014)^63^, Kraal (2017)^75^ |  |  |
| **Lack of tailored exercise prescription** (including insufficient progression of exercise) | HEART mHealth: Maddison (2015)^76^, TRiCH: Avila (2020)^77^ | TRiCH: Avila (2020)^77^ | Dale (2015)^49^, Snoek (2019)^50^, Snoek (2020)^50^ |  |
| **Lack of relapse prevention mechanisms** |  |  | Dale (2015)^49^ |  |
| **Factors related to clinician-patient relationship** |  |  |  |  |
| **Unmonitored exercise** |  |  | Reid (2012)^67^ |  |
| **Passive intervention** (e.g. education content available on a website) | Dale (2015)^49^, HEART mHealth: Maddison (2015)^76^, Kayser(2019)^60^ |  | Reid (2012)^67^, HEART mHealth: Maddison (2015)^76^, Pfaeffli Dale (2015)^64^, Maddison (2014)^62^, Kayser(2019)^60^ | Kayser(2019)^60^ |
| **Influence of patients' preference on the choice of modality of care** (including the perception of lack of usefulness) | Kayser(2019)^60^ |  | REMOTE-CR: Rawstorn (2018)^41^, Maddison (2019)^19^, EU-CaRE: Snoek (2019)^50^, Snoek (2020)^50^, TRiCH: Avila (2018)^54^, Avila (2020)^77^ (increasing drop-outs), Kayser(2019)^60^ | Kayser(2019)^60^ |
|  |  |  |  |  |
|  |  |  |  |  |
| **Reduced contact with intervention** (including lack of follow-up, maintenance) |  |  | Devi (2014)^48^, Varnfield (2014)^43^ |  |
| **No control of other medical conditions** (eg. Muskuloeskeletal conditions) |  |  | Telerehab-III: Frederix (2015)^46,78^, Frederix (2016)^65^, HEART mHealth: Maddison (2015)^76^, Pfaeffli Dale (2015)^64^, Maddison (2014)^62^, TRiCH: Avila (2020)^77^ |  |
| **Patients' previous sedentary behaviour** | TRiCH: Avila (2018)^54^ (stepcount), Kayser(2019)^60^, Neubeck (2011)^53^ |  | Kayser(2019)^60^ |  |
