# Supplementary figures and images for: Key features in telehealth-delivered cardiac rehabilitation required to optimize cardiovascular health in coronary heart disease: a systematic review and realist synthesis
Source: Eur Heart J Digit Health. 2024 Jan 5;5(3):208–18. doi: 10.1093/ehjdh/ztad080 (PMC11104477; doi:10.1093/ehjdh/ztad080)

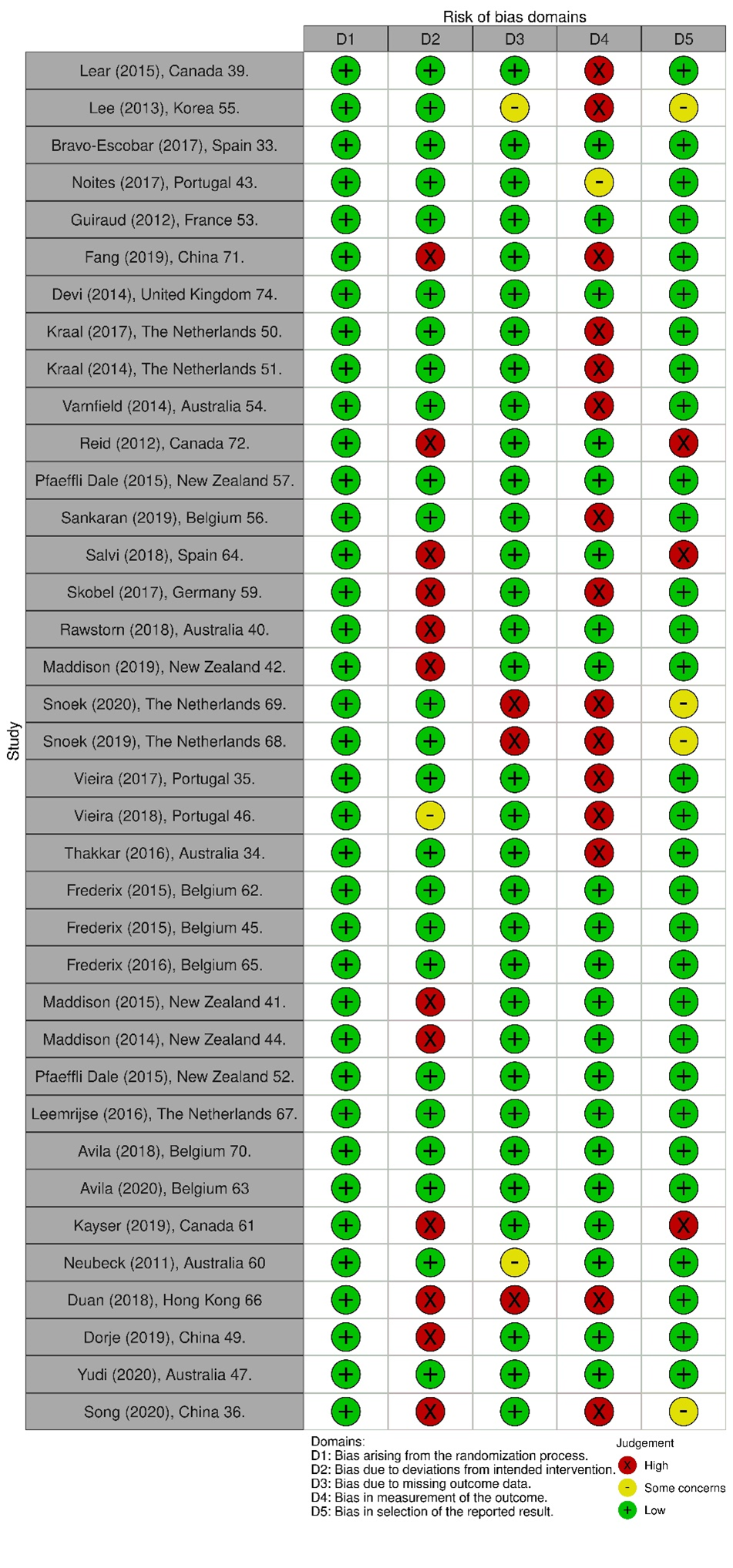

Supplement: ztad080_Supplementary_Data [file ztad080_supplementary_data.zip › GallegosRejas_Supplementary_Figure_1_RiskofBias.tif]
